# P-2076. COVID-19 Vaccination Patterns and Timing among Privately Insured Pregnant Persons, 2020-2023

**DOI:** 10.1093/ofid/ofae631.2232

**Published:** 2025-01-29

**Authors:** Lakshmi Panagiotakopoulos, Amadea Britton, Ruth Link-Gelles, Wafa Tarazi, Alexandra Stone, Nwanneamaka Ume, Andrea Steffens, Katherine Andrade, Garrett Gremel, Ami R Buikema, Wenya Grace Yang

**Affiliations:** Centers for Disease Control and Prevention, Atlanta, Georgia; Centers for Disease Control and Prevention , Atlanta, GA; Centers for Disease Control and Prevention, Atlanta, Georgia; Optum Serve, La Crosse, Wisconsin; OSC, Washington, District of Columbia; Optum, La Crosse, Wisconsin; OPTUM, Eden Prairie, Minnesota; Optum, La Crosse, Wisconsin; Optum, La Crosse, Wisconsin; Optum, La Crosse, Wisconsin; Optum, La Crosse, Wisconsin

## Abstract

**Background:**

Pregnant people with COVID-19 are at higher risk of severe illness, pregnancy complications, and adverse pregnancy outcomes. While maternal COVID-19 vaccination has been shown to be safe and effective for pregnant people and their infants, vaccine coverage rates during pregnancy are not optimal. Additional data on the receipt and distribution of COVID-19 vaccine prior to and during pregnancy can inform efforts to increase coverage. We describe COVID-19 vaccination patterns and timing of the most recent vaccine dose among pregnant people.

**Figure 1.**
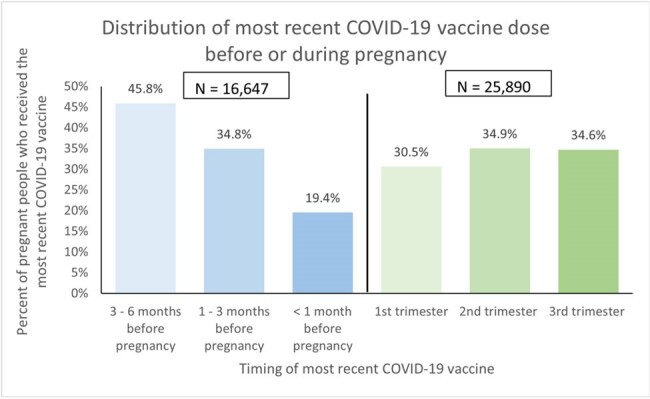
Distribution of COVID-19 Vaccination Patterns Prior to and During Pregnancy among Privately Insured Pregnant Persons, December 2020 - July 2023 Notes: In 25,890 pregnancy episodes, the most recent COVID-19 vaccination was received during pregnancy and vaccinations were approximately evenly distributed across trimesters. In the remaining 16,647 episodes, the most recent vaccination was received before the start of pregnancy with almost half of the vaccinations occurring 3-6 months before pregnancy.

**Methods:**

Vaccination data from a large national commercial claims database were supplemented with data from 21 Immunization Information Systems for which we have permission to use data. We calculated the percentage of pregnant people ages 18-55 years identified between June 2020 and July 2023 who received a COVID-19 vaccination between December 2020 and July 2023. We describe timing of most recent dose received in the 6 months prior to pregnancy (3-6 months, 1-3 months, < 1 month before last menstrual period [LMP]) and during pregnancy, by trimester (first, second, third trimester).

**Results:**

This study included a total of 120,633 pregnancies among 102,311 pregnant people (mean age at LMP = 32 years; standard deviation: 5). About a third (35.3%, n = 42,537) received ≥1 COVID-19 vaccine dose within the 6 months prior to or during pregnancy; of those, 39.1% received ≥1 dose prior to pregnancy, 41.6% received ≥1 dose during pregnancy, and 19.3% received doses both before and during pregnancy. Among those who received the vaccine before pregnancy, 45.8% received the most recent dose 3-6 months, 34.8% 1-3 months, and 19.4% < 1 month before LMP. Receipt of the most recent COVID-19 vaccine dose during pregnancy was distributed almost evenly across the first (30.5%), second (34.9%), and third (34.6%) trimesters.

**Conclusion:**

This study found that approximately one third of pregnancies included people who were vaccinated with at least 1 dose of COVID-19 vaccine during pregnancy or within 6 months prior to LMP. The most recent dose was not received more frequently at any time before or during pregnancy. Understanding COVID-19 vaccination patterns and timing is important to assess adherence to vaccination recommendations among this high-risk population.

**Disclosures:**

Wafa Tarazi, PhD, MHPA, UnitedHealth Group: I am an employee at UHG alexandra stone, PhD, United Health Group: Stocks/Bonds (Public Company) Nwanneamaka Ume, MPH, Optum: I am an Employee of Optum, a UnitedHealth Group subsidiary Andrea Steffens, MPH, OPTUM: Employee|UnitedHealth Group: Stocks/Bonds (Public Company) Katherine Andrade, MPH, Optum: Employee|United Health Group: Stocks/Bonds (Public Company) Garrett Gremel, MS, Optum: Employee Ami R. Buikema, MPH, Optum: Employee|UnitedHealth Group: Stocks/Bonds (Public Company) Wenya Grace Yang, MPA, MA, UnitedHealth Group: I am an employee of UHG.|UnitedHealth Group: Stocks/Bonds (Public Company)

